# Evaluation of a new self‐administered digital test battery for timely and accurate detection of cognitive impairment (BraincheX)

**DOI:** 10.1002/alz70856_107240

**Published:** 2026-01-08

**Authors:** Sophia Rutt, Carolin Isabella Kurz, Robert Perneczky, Boris‐Stephan Rauchmann, Lena Burow, Marleen Taute, Paulina Tegethoff

**Affiliations:** ^1^ Department of Psychiatry and Psychotherapy, LMU Hospital, LMU Munich, Munich, Bavaria, Germany; ^2^ Department of Psychiatry and Psychotherapy, LMU Hospital, LMU Munich, Munich, Germany; ^3^ Department of Psychiatry and Psychotherapy, University Hospital, LMU Munich, Munich, Germany; ^4^ Department of Psychiatry and Psychotherapy, University Hospital, LMU Munich, Munich, Bavaria, Germany; ^5^ LMU University Hospital, Munich, BY, Germany

## Abstract

**Background:**

Timely diagnosis of Alzheimer's disease (AD) using cognitive assessments is crucial for effective intervention. Standardized tests such as the CERAD‐Plus battery aid in diagnosing decline and treatment eligibility. Digital test batteries may offer a streamlined alternative by assessing cognitive flexibility, processing speed, memory, and abstraction in a self‐paced, non‐verbal format. BraincheX was developed to provide a pragmatic, accurate means of cognitive testing in diverse settings; it includes digital adaptations of sensitive paper‐and‐pencil tests, and a new visual memory test (VMT). This pilot study examines the accuracy of BraincheX in detecting cognitive decline in a convenience memory clinical sample.

**Method:**

Participants were recruited from an academic memory clinic and categorized into four groups based on amyloid positivity and clinical expert consensus, defined as AD spectrum (dmild ementia, *n* = 12; MCI, *n* = 10), subjective decline (*n* = 17), and amyloid‐negative healthy controls (*n* = 10). Corresponding variables from BraincheX and CERAD‐plus were directly compared through correlation analysis. Comparisons between diagnostic groups were conducted using ANOVA. Each subtest was standardized to z‐scores and overall scores were calculated. Optimal cut‐off values were defined using ROC analysis.

**Result:**

Preliminary results indicate strong correlations between corresponding variables on BraincheX and CERAD‐plus (TMT‐B r=.498, *p* <.001, *n* = 47; Clock Drawing Test r=.592, *p* <.001, *n* = 40). Group differentiation analyses confirmed BraincheX's effectiveness in distinguishing between diagnostic groups (VMT F(2)=6.674, *p* = .003, η2=.233; TMT‐B F(2) =13.327, *p* <.001, η2=.367; Clock Test F(2)=6.768, *p* = .003, η2=.227). BraincheX total scores were strongly correlated with CERAD‐pluse total scores (r=.762, *p* <.001). Diagnostic accuracy analysis using ROC curves demonstrated a high level of discrimination (AUC=.884). The optimal cut‐off score maximizing sensitivity and specificity distinguishing between AD and Controls resulted in 92% sensitivity and 86.4% specificity.

**Conclusion:**

Initial results support BraincheX's ability to discriminate between diagnostic groups and its correlation with CERAD scores. Limitations include a small sample size and potential bias due to the participants' limited familiarity with digital tools. These findings suggest BraincheX has potential as a digital screening tool. Combined with other biomarkers, it may help identify patients for disease‐modifying treatments. Further validation with larger, diverse samples is ongoing.

